# Epidemiological Characteristics of Parainfluenza Virus Type 3 and the Effects of Meteorological Factors in Hospitalized Children With Lower Respiratory Tract Infection

**DOI:** 10.3389/fped.2022.872199

**Published:** 2022-04-27

**Authors:** Ming Xu, Wei Yue, Xinyue Song, Luyao Zeng, Li Liu, Jinwei Zheng, Xiaofang Chen, Fangfang Lv, Shunhang Wen, Hailin Zhang

**Affiliations:** ^1^Department of Pediatric Pulmonology, Second Affiliated Hospital and Yuying Children's Hospital, Wenzhou Medical University, Wenzhou, China; ^2^Department of Pediatrics, First Affiliated Hospital of Xi'an Jiaotong University, Xi'an, China; ^3^Clinical Research Center, Affiliated Eye Hospital of Wenzhou Medical University, Wenzhou, China

**Keywords:** HPIV-3, meteorological factors, epidemiological characteristics, GAM, ARIMA, LASSO

## Abstract

**Objective:**

To investigate the relationship between meteorological factors and Human parainfluenza virus type 3 (HPIV-3) infection among hospitalized children.

**Methods:**

All hospitalized children with acute lower respiratory tract infections were tested for viral pathogens and enrolled, at the second affiliated hospital of Wenzhou medical university, between 2008 and 2017. Meteorological data were directly obtained from Wenzhou Meteorology Bureau's nine weather stations and expressed as the mean exposure for each 10-day segment (average daily temperatures, average daily relative humidity, rainfall, rainfall days, and wind speed). The correlation between meteorological factors and the incidence of HPIV-3 was analyzed, with an autoregressive integrated moving average model (ARIMA), generalized additive model (GAM), and least absolute shrinkage and selection operator (LASSO).

**Results:**

A total of 89,898 respiratory specimens were tested with rapid antigen tests, and HPIV-3 was detected in 3,619 children. HPIV-3 was detected year-round, but peak activities occurred most frequently from March to August. The GAM and LASSO-based model had revealed that HPIV-3 activity correlated positively with temperature and rainfall day, but negatively with wind speed. The ARIMA (1,0,0)(0,1,1) model well-matched the observed data, with a steady R^2^ reaching 0.708 (Ljung-Box Q = 21.178, *P* = 0.172).

**Conclusion:**

Our study suggests that temperature, rainfall days, and wind speed have significant impacts on the activity of HPIV-3. GAM, ARIMA, and LASSO-based models can well predict the seasonality of HPIV-3 infection among hospitalized children. Further understanding of its mechanism would help facilitate the monitoring and early warning of HPIV-3 infection.

## Introduction

Human parainfluenza virus (HPIVs) is the common cause of acute respiratory infections. HPIV-3 accounts for the majority of HPIV infections in infants and young children (1). In Australia, HPIV-3 incidence was estimated at over 20% for infants in the first year of life (2). Almost all children (97%) under 5 years old showed seropositivity for HPIV-3 (3). Evidence is emerging that meteorological conditions, particularly temperature may influence HPIVs epidemics (4). It is also possible that weather patterns may facilitate HPIV transmission by affecting duplication and transmission of viruses, as well as population immune status (5). The autoregressive integrated moving average (ARIMA) model and generalized additive model (GAM) were suitable for the time-series analysis of nonlinear meteorological factors, with improved accuracy for model outputs (6). The least absolute shrinkage and selection operator (LASSO) was not affected by correlations between predictors, and effectively eliminated redundancy, which improved the selection quality (7). We aimed to explore the correlations between meteorological factors and incidence of HPIV-3 among hospitalized children, with ARIMA-, GAM-, and LASSO- based models.

## Materials and Methods

### Subject Information

We recruited all hospitalized children with lower respiratory tract infection (LRTI), including those admitted to the PICU and NICU, from January 2008 to December 2017. All procedures were performed in accordance with the ethical standards of the institutional and national research committee as well as the 1964 Helsinki declaration and its later amendments. Our study procedures were approved by the ethics committee of the second affiliated hospital of Wenzhou medical university (No: LCKY2019-234).

### Methods for Virus Detection

Nasopharyngeal aspirate (NPA) and/or sputum samples were routinely collected from hospitalized children with LRTI. These specimens were tested for common viral pathogens (respiratory syncytial virus, adenovirus, influenza A, influenza B, HPIV-1, HPIV-2, and HPIV-3), at the clinical laboratory of the second affiliated hospital of Wenzhou medical university, within 6 h, with direct immunofluorescence (DIF), using the D3^®^ Ultra™ DFA Respiratory Virus Screening & ID Kit (Diagnostic Hybrids, Inc. Athens, OH), strictly following the manufacturer's instruction. Patients showing positive results for HPIV-3 antigen were considered an infection. Test results were directly exported from our database. Samples acquired within 2 weeks from the same patients were considered to be within the same course of the disease.

### Meteorological Data

Meteorological data for Wenzhou were obtained directly from nine major weather stations of the Wenzhou Climatic Bureau, including average temperature (°C), relative humidity (RH%), rainfall (mm), and rainfall days (d). One weather station is located in an urban area, and the other eight stations in each county around the hospital, which is situated in the center of Wenzhou. Daily data for the first, middle, and late 10 days of the month were summarized in segments and migrated into a 10-day average.

### Statistical Analysis

HPIV-3 detection rate (%) was defined as the percentage of positive test results for HPIV-3 each month. The Chi-square test (χ^2^) was used to compare the positive rates among different age groups. Meteorological data (mean ± SD) was used as independent variables. ARIMA and LASSO regression models were used to explore their correlations with HPIV-3 detection rates. All statistical analyses were performed with R (R Foundation for Statistical Computing, Vienna, Austria). The “forecast,” “tseries,” “mgcv,” and “lars” R packages were used. *P* < 0.05 were considered statistical significance.

## Results

### The HPIV-3 Detection Rate of Age

Of the 89,898 patients, 3,619 (4%) of which were positive for HPIV-3. Patients under 1-year-old showed the highest detection rate (5.3%, 2203/41451). 4.4% (974/22109) of the patients between 1-year-old and 3-year-old were tested positive for HPIV-3, and only 1.7% (442/26338) in patients older than 3-year-old. The HPIV-3 infection rate seemed to decrease with age. Significant differences were noted (*P* < 0.01, χ^2^ = 562.2).

### Seasonality of HPIV-3 Incidence and ARIMA Model With Meteorological Factors

**Figure 1:**
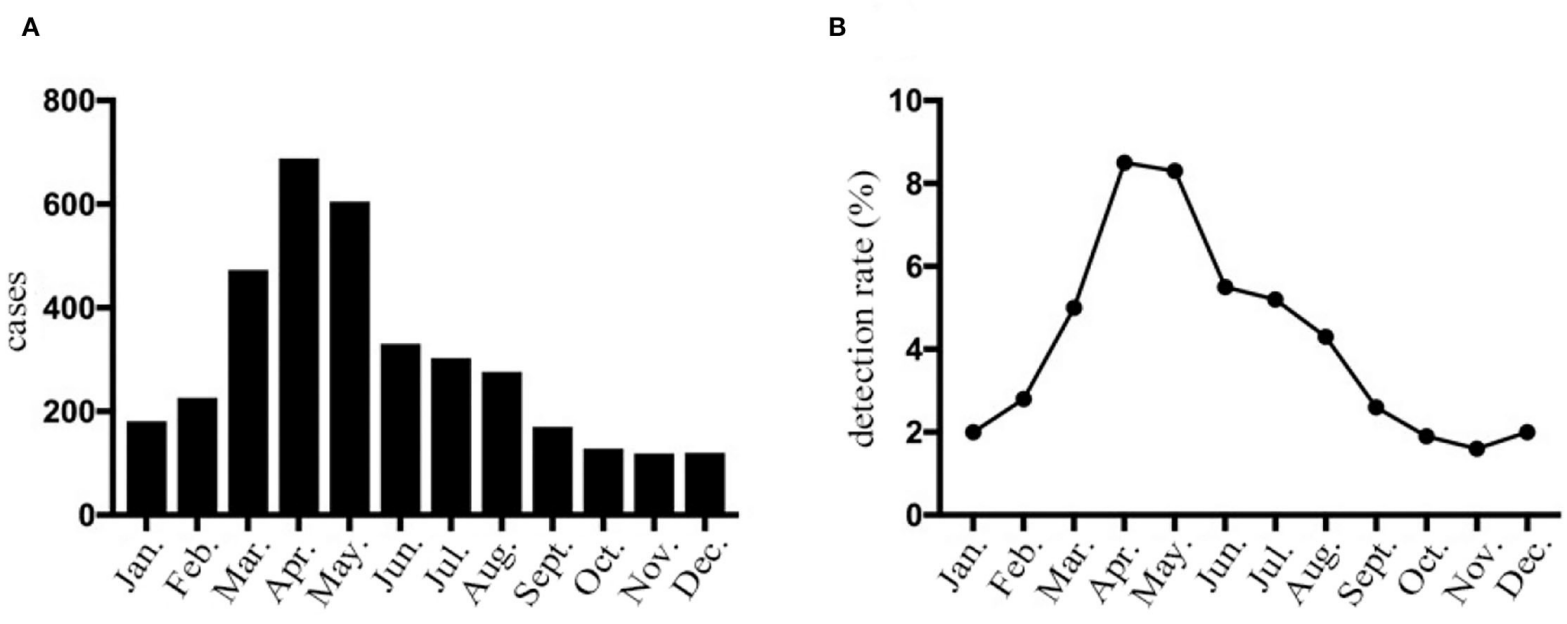
HPIV-3 detection rates of hospitalized children with LRTI in Wenzhou by month. **(A)** The number of HPIV-3 cases collected in hospitalized children with lower respiratory tract infection according to month. **(B)** Prevalence of HPIV-3 infections in hospitalized children with lower respiratory tract infection according to month.

**Figure 2:**
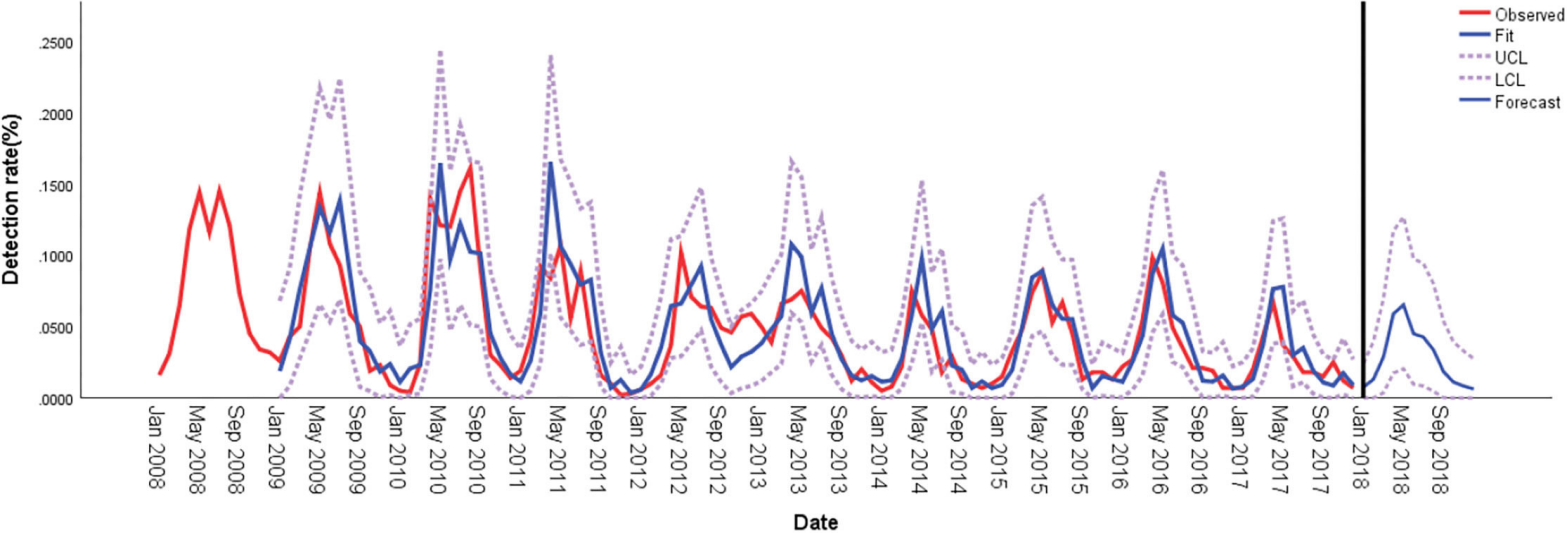
Comparison of HPIV-3 detection rates predicted using ARIMA and observed values in hospitalized children in Wenzhou, China. ARIMA, autoregressive integrated moving average; LCL, lower confidence limit; UCL, upper confidence limit.

The seasonal distribution revealed that HPIV-3 occurred throughout the year, with a single peak during Spring and Summer, especially between April and May (Figure 1). Overall, the detection rate of HPIV-3 decreased with year. Our ARIMA (1,0,0)(0,1,1) model predicted a seasonal trend in HPIV-3 detection rate, which was significantly higher in Spring and Summer. Such result well matched the observed data, with a steady R^2^ reaching 0.708 (Ljung-Box Q = 21.178, *P* = 0.172) (Figure 2).

### Association Between HPIV Epidemics and Meteorological Factors

#### Mean Values of Meteorological Parameters

The meteorological data of Wenzhou from 2008 to 2017 indicated that the average temperature was 18.37 ± 7.21°C, the average relative humidity was 76.95 ± 5.87%, the average rainfall was 144.76 ± 100.01 mm/month, the average rainfall days was 14.12 ± 5.00 days/month, and the average wind speed was 2.01 ± 0.26 m/s.

#### Generalized Additive Model

**Figure 3:**
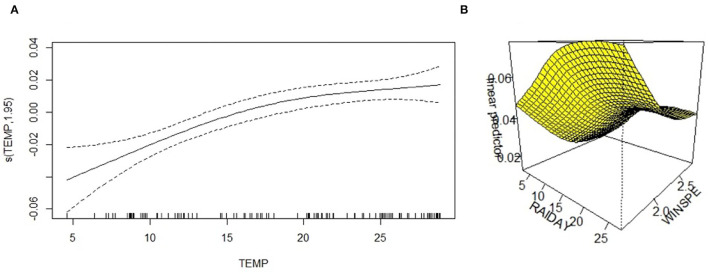
A three-dimensional surface-fitting graph of the relationship between HPIV-3 detection rate and average temperature, precipitation days, and average wind speed; **(A)** The relationship between HPIV-3 detection rate and average temperature was nearly linearly. **(B)** Three-dimensional surface-fitting graph of the relationship between HPIV-3 detection rate, rainfall days and average windspeed using GAM; WINSPE, windspeed.

All meteorological factors were associated with the detection rate of HPIV-3 in the univariate analysis. In the multivariate analysis, average temperature was nearly linearly associated with HPIV-3 detection rate (Table 1, Figure 3A), rainfall days was positively associated with HPIV-3 detection rate. The HPIV-3 detection rate decreased as wind speed increased (Figure 3B).

**Table 1:** HPIV-3 detection rate according to meteorological factors using the generalized additive model (GAM).

**Variable**	**Univariate analysis**			**Multivariate analysis**		
	**df**	**F**	***P***	**df**	**F**	***P***
Average temperature (°C)	1.952	13.630	<0.001	1.000	5.193	<0.001
Average relative humidity (%)	1.000	24.580	<0.001			–
Rainfall (mm)	2.352	10.620	<0.001			–
Days of rainfall (d)	1.000	21.270	<0.001	2.074	2.838	0.039
Average wind speed (m/s)	3.952	2.954	0.018	1.796	3.463	0.028

*df, degree of freedom*.

#### LASSO-Based Models

**Figure 4:**
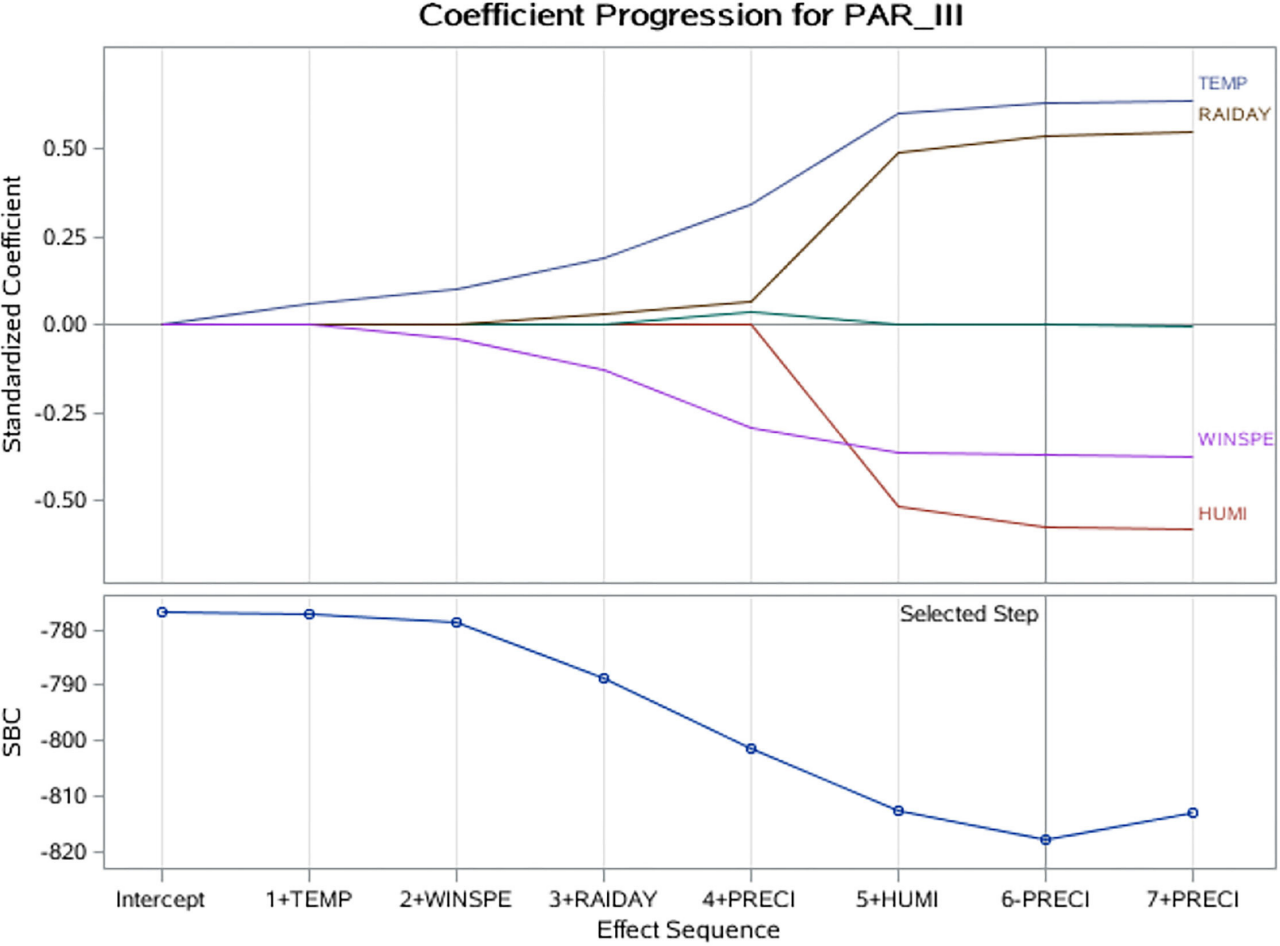
LASSO coefficient progression plot of the HPIV-3 detection rate. HUMI, humidity; LASSO, least absolute shrinkage and selection operator; PRECI, precipitation (rainfall); RAIDAY, days of rainfall; SBC, Schwarz–Bayes criterion; TEMP, temperature; WINSPE, windspeed.

The LASSO regression model revealed that HPIV-3 detection rate was positively associated with average temperature and rainfall days. Average relative humidity and wind speed were negatively associated with HPIV-3 detection rate (Table 2, Figure 4).

**Table 2:** Correlation between HPIV-3 detection rate and meteorological factors according to least absolute shrinkage and selection operator (LASSO)-based models.

**Indicators**	**Variables**	**Estimated coefficient**	**Standard estimate coefficient**
HPIV-3 detection rate	Average temperature	0.003385	0.631025
	Average relative humidity	0.003782	0.57351
	Days of rainfall	0.004148	0.536592
	Average wind speed	0.055465	0.372052

## Discussion

With DIF, the detection rate of HPIV detection rate was 4.7%(4176/89898), accounting for the second most common pathogen. The detection rates for HPIV-1, HPIV-2, and HPIV-3 were 0.61%(550/89898),0.086%(74/89898), and 4.03%(3619/89898), respectively. Such results suggested that HPIV-3 was one of the major viral pathogens in Wenzhou, whereas HPIV-1 and HPIV-2 were relatively rare, which was consistent with the previous study (8). HPIV-3 infection was considered common among infants and young children, and HPIV-1 and HPIV-2 infections were more common among older children (9). Villaran et al. (10) showed HPIV-3 detection rate was 4 times higher in children under 5 years old than in those aged over 5.

Detection of all 3 subtypes of HPIV was distributed across all age groups but majorly occurred in children 10 years old or younger, the detection rate decreased with age. Children under 6 months old had the highest HPIV-3 detection rate (4.7%), and children aged 10 to 18 years old had the lowest detection rate (0.8%). Such a result was in line with previous studies (11, 12).

The HPIV-3 epidemic resulted in a global health burden with seasonal and regional differences. Data from Japan (13), Korea (12), North America (14), and Europe (15) had suggested that HPIV-3 exhibited a Spring-Summer seasonality. Howard et al. (16) found that the highest prevalence of HPIV-3 occurred in the Summer. Data from the National Respiratory and Enteric Virus Surveillance System suggested that peaks of HPIV-3 infection were observed annually during Spring and Summer, with smaller peaks in the fall occurring at irregular intervals (17). In Jaipur, western India, HPIV-3 was found to be circulating in the index population during all 3 years, predominantly during the post-monsoon and winter seasons (18). Rio de Janeiro found that the HPIV-3 cycle began in autumn and peaked in the Spring of a 28 years study (19). A systematic review (4) suggested that HPIV infections peaked during Spring and early Summer in both hemispheres. Given the lack of annual laboratory-confirmed data for HPIV, global reports on its seasonality remained sparse.

In consistency with the data from other cities in China, we observed that peaks of HPIV-3 infection in Wenzhou occurred in Spring and Summer as well (8, 11, 20). This study had a large sample size of 10 years, our analysis contributed information about HPIV-3 seasonality and its correlation with meteorological factors.

The exact mechanism of HPIV-3 seasonality remained unclear, and it's plausible that meteorological factors, including temperature, humidity, rainfall, sunshine, and wind speed, were the major causes (4). Furthermore, exposure to infection, virus survival rate, and population immune status affected the prevalence of the virus as well (21). Temperature appeared to be the main meteorological factor responsible for the seasonality of respiratory viruses. Generally, the lower temperature was considered to promote virus duplication, however, HPIV-3 preferred higher temperatures (15). Chen et al. (22) reported an association between temperature and HPIV-3 detection rate in children hospitalized for respiratory infection, with ARIMA and generalized linear model (GLM). In this study, LASSO-based models found a correlation between HPIV-3 detection rate and temperature, rainfall days, and wind speed. GAM analysis showed an almost linear correlation between temperature and HPIV-3 detection rate. Interestingly, HPIV epidemics were reported, in many studies from different countries with different climate characteristics, to have the same regular annual seasonal peak activity period between Spring and Summer. However, the studies cited in this article were mainly coming from tropical, subtropical, and temperate climate regions. Moreover, HPIV epidemics seem to demonstrate a clearer Spring-Summer peak in temperate settings and less pronounced in studies from tropical settings. Such a phenomenon may be explained by higher annual average temperatures in tropical regions. Thus, we suppose temperature may be the main meteorological factor for the HPIV-3 epidemic. We found that the HPIV-3 detection rate increased with rainfall days, but decreased with wind speed. Yan et al. (20) showed that the HPIV-3 detection rate in outpatients was also positively associated with temperature and rainfall. Though not fully understood, HPIV-3 aerosols have been presumed to favor its survival and enhance its transmission due to the higher humidity on rainy days. Additionally, rainfall increased the risk of virus exposure with increased indoor contact (23). However, Price et al. (15) reported a negative association between humidity and HPIV-3 detection rate, they believed that HPIV-3 can tolerate dry climates. However, recent studies detected an inconsistent correlation between the HPIV epidemic and rainfall days (24, 25). Further studies were required. Our study found that HPIV-3 detection rates were negatively associated with wind speed. We presumed that higher wind speed reduced the adhesion to the skin and nasal mucosa, however, a more persuasive explanation for its mechanisms requires further studies.

We used meteorological factors (temperature, relative humidity, rainfall, rainfall days, and wind speed) as independent variables to analyze the detection rate of HPIV-3 among hospitalized children. ARIMA models well predicted HPIV-3 incidence. We used GAM to visualize the effects of meteorological factors, and corrected collinearity with LASSO-based models. The use of multiple statistical models during time series analysis reduced bias (26), and similar conclusions from different models further confirmed the correlation between meteorological factors and HPIV-3 detection rate.

However, this study had limitations. Firstly, no lab result can guarantee infection. PCR kits for HPIV were launched in China in April 2019, and not be available at our hospital until 2020. Test for antibody tiers was not available in our hospital. Thus, DIF had been the only method we applied to all hospitalized children throughout the 10 years study period. However, DIF had high specificity, positive results could be persuasive. Colonization was also a noteworthy concern. As for HPIV, a case-control study conducted by Bhuiyan et al. (27) showed that HPIV was the only respiratory virus that had not been detected among 230 healthy children. Such a result may suggest that HPIV colonization was not common among children. Secondly, as mentioned above, DIF had high specificity but low sensitivity, so the actual infection rate of HPIV-3 might be underestimated. As a retrospective study, using DIF positive results solely to confirm HPIV infection does have certain limits, further studies involving multiple detection methods and their comparisons should be carried out. Thirdly, though HPIV epidemics were reported, in many reports from different countries with different climate characteristics, to have the same regular annual seasonal peak activity period between Spring and Summer, our single-centered retrospective study didn't provide enough evidence to explain its mechanisms. Fourthly, all participants recruited were hospitalized children, it's unclear whether the conclusion could be extrapolated to the population. Further study recruiting healthy controls was needed. Lastly, the associations observed here do not imply causal mechanisms.

In conclusion, we observed an annual peak in Spring and Summer for the HPIV-3 epidemic in Wenzhou. Our study suggests that temperature, rainfall days, and wind speed have significant impacts on the activity of HPIV-3. GAM, ARIMA, and LASSO-based models can well predict the seasonality of HPIV-3 infection among hospitalized children. The exact mechanism remains unclear. Possible explanations with meteorological factors may be presented as follows. (1) HPIV-3 prefers higher temperatures, the ability to replicate stably promoted its transmission in Spring and Summer, while replication of other viruses was suppressed. (2) Higher humidity enhances aerosol transmission. (3) Rainfall increased humidity and may be an increased tendency for individuals to seek shelter during rain events. Thus, increasing the probability of exposure to indoor spaces where human density may be higher and conditions optimal for droplet and fomite transmission. Further understanding of its mechanism would help facilitate the monitoring and early warning of HPIV-3 infection.

## Data Availability Statement

The raw data supporting the conclusions of this article will be made available by the authors, without undue reservation.

## Ethics Statement

The studies involving human participants were reviewed and approved by the Ethics Committee of the Second Affiliated Hospital and Yuying Children's Hospital, Wenzhou Medical University. Written informed consent to participate in this study was provided by the participants' legal guardian/next of kin.

## Author Contributions

XS and LZ participated in project execution and data collection. LL, JZ, and XC completed data analysis. MX and WY are responsible for project writing/execution and article writing. FL, SW, and HZ gave guidance on this subject and revised the article. HZ supervised the whole project. All authors contributed to the article and approved the submitted version.

## Conflict of Interest

The authors declare that the research was conducted in the absence of any commercial or financial relationships that could be construed as a potential conflict of interest.

## Publisher's Note

All claims expressed in this article are solely those of the authors and do not necessarily represent those of their affiliated organizations, or those of the publisher, the editors and the reviewers. Any product that may be evaluated in this article, or claim that may be made by its manufacturer, is not guaranteed or endorsed by the publisher.
